# The S-S.M.A.R.T: A New Prognostic Tool for Patients with Suspected Sepsis in the Emergency Department

**DOI:** 10.1155/2023/8852135

**Published:** 2023-08-10

**Authors:** Ye Jin Kim, Jong Won Kim, Kyeong Ryong Lee, Dae Young Hong, Sang O Park, Young Hwan Lee, Sin Young Kim

**Affiliations:** ^1^Department of Emergency Medicine, Konkuk University Medical Center, Seoul, Republic of Korea; ^2^Department of Emergency Medicine, Konkuk University School of Medicine, Seoul, Republic of Korea

## Abstract

**Background:**

The sepsis screening tool is essential because it enables the rapid identification of high-risk patients and facilitates prompt treatment. Quick Sequential Organ Failure Assessment (qSOFA) is a widely used screening tool for sepsis. However, it has limitations in predicting patient prognosis. We developed the S-S.M.A.R.T (*s*epsis evaluation with *s*hock index, *m*ental status, *a*ge, and *ROX* index on *t*riage) and aimed at evaluating it as a screening tool for patients with suspected sepsis in the emergency department.

**Methods:**

We conducted a single-center retrospective chart review of patients with suspected sepsis in the emergency department. We compared the prognosis prediction abilities of the S-S.M.A.R.T and qSOFA scores in patients with suspected sepsis. The primary outcome was 7-day mortality, and the secondary outcomes included 30-day mortality and ICU admission. The receiver operating characteristic (ROC) curve analysis and the chi-square test were used.

**Results:**

In total, 401 patients were enrolled. The mean age of the patients was 72.2 ± 15.6 years, and 213 (53.1%) of them were female. The S-S.M.A.R.T had superior predictive ability for prognosis of patients with suspected sepsis compared to qSOFA (area under the ROC curve (AUC) of 0.789 vs. 0.699; *p*=0.02 for 7-day mortality, AUC of 0.786 vs. 0.681; *p* < 0.001 for 30-day mortality, AUC 0.758 vs 0.717; *p*=0.05 for ICU admission).

**Conclusion:**

The S-S.M.A.R.T can be useful in predicting the prognosis of patients with suspected sepsis in the emergency department.

## 1. Introduction

Sepsis is a potentially life-threatening medical condition caused by a deleterious host response to infection, and prompt medical attention and treatment are necessary [1]. Therefore, the early identification of sepsis in patients with suspected infection is crucial for improving patient outcomes [2]. Screening tools for sepsis can help physicians identify serious patients and immediately initiate appropriate treatment to prevent a poor prognosis [3, 4]. International guidelines for the management of sepsis and septic shock recommend screening patients at a high risk for sepsis [2]. The emergency department (ED) is often the first point of contact for patients with sepsis. Therefore, these patients must be screened in the ED to improve outcomes and reduce mortality rates.

Clinical tools such as the systemic inflammatory response syndrome score, sequential organ failure assessment (SOFA), national early warning score, and modified early warning score are used for sepsis screening [4]. The quick sequential organ failure assessment (qSOFA), which comprises three clinical variables (altered mental status, low blood pressure, and rapid respiratory rate), is also a sepsis screening tool [1]. It has the advantage of being simple and easy to use. Because the qSOFA can be performed quickly and easily at the bedside, it is widely used for triaging febrile patients in the ED. Although the qSOFA is a simple and easy-to-use screening tool for sepsis in the ED, it has some limitations, including low sensitivity in identifying patients with sepsis [5–7]. In addition, several studies have yielded inconsistent findings regarding the usefulness of the qSOFA as a predictor of poor prognosis in patients with sepsis.

Simple screening tools such as qSOFA that are more accurate in predicting severity would be useful for emergency physicians. We developed the S-S.M.A.R.T (*s*epsis evaluation with *s*hock index, *m*ental status, *a*ge, and *ROX* index on *t*riage) to replace the qSOFA for sepsis screening in the ED and aimed at evaluating its effectiveness as a screening tool for predicting the severity of disease in patients with suspected infections in the ED. We assessed the clinical utility of the S-S.M.A.R.T in predicting severity in patients with suspected infection in the ED and compared its performance with that of qSOFA.

## 2. Methods

This study was conducted at a tertiary hospital in Seoul, Korea, from November 2021 to December 2022. We retrospectively reviewed the electronic medical records of patients >18 -years-old who were admitted to our hospital via the ED with a diagnosis of an infectious disease. Patients who presented to the ED with cardiac arrest, those transferred from other hospitals, and those with missing outcome information were excluded. This study was approved by the Institutional Review Board (KUMC2023-04-015), and the requirement for informed consent was waived.

All patients who visited the ED were triaged by a nurse, and their vital signs, including systolic blood pressure, diastolic blood pressure, heart rate, respiratory rate, body temperature, oxygen saturation (SpO_2_), level of consciousness, and fraction of inspired oxygen (FiO_2_), were measured at the time of presentation. In addition, the patients were classified according to the Korean Triage and Acuity Scale. Patients with suspected sepsis received the appropriate treatment in accordance with the 1-hour sepsis bundle recommendations. Empirical broad-spectrum antibiotics were administered within 1 hour, and fluid resuscitation and vasopressors were implemented as necessary.

The S-S.M.A.R.T score was determined using four components: shock index (heart rate/systolic blood pressure), mental status, age, and ROX index (SpO_2_/FiO_2_/respiratory rate) (Table 1). We calculated the qSOFA and S-S.M.A.R.T scores based on data from the electronic medical records. We also obtained clinical data including patient age, sex, medical history, diagnosis, comorbidities, length of hospital stay, mechanical ventilation, ICU admission, 7-day mortality, and 30-day mortality.

The primary outcome measure in our study was 7-day mortality, whereas the secondary outcomes were 30-day mortality and ICU admission. We used the receiver operating characteristic (ROC) curve analysis to evaluate the effectiveness of various screening tools (qSOFA and S-S.M.A.R.T) in predicting the severity of infection. In addition, we used the chi-square test to compare categorical variables, which are presented as numbers and percentages. Statistical analyses were performed using the SPSS 28 (version 28.0, Seoul, Korea) and MedCalc 22 (MedCalc Ltd., Mariakerke, Belgium) software packages. Statistical significance was set at *p* ≤ 0.05.

## 3. Results

**Figure 1:**
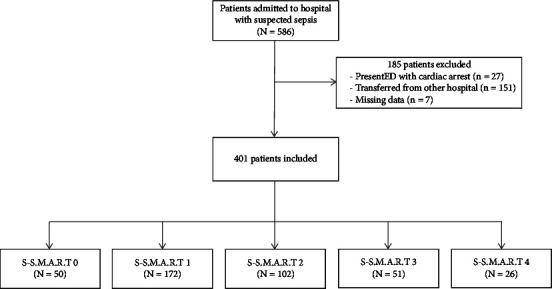
Flowchart of the study population. Abbreviations: S-S.M.A.R.T (sepsis evaluation with shock index, mental status, age, and ROX index on triage).

A total of 401 patients were included in this study. (Figure 1) The average age of the patients was 72.2 years, with a standard deviation of 15.6 years. Among them, 213 (53.1%) were women. The overall mortality rate observed in this study was 16.2%. 158 (39.7%) patients had positive blood cultures. The 7-day mortality was 16 (10.1%) for blood culture positive patients and 14 (5.8%) for blood culture negative patients (*p*=0.082), while the 30-day mortality was 22 (13.8%) for former and 32 (13.2%) for latter (*p*=0.486). There was no statistically significant association between bacteremia and the mortality rate. The baseline characteristics of the study participants are presented in Table 2.

**Figure 2:**
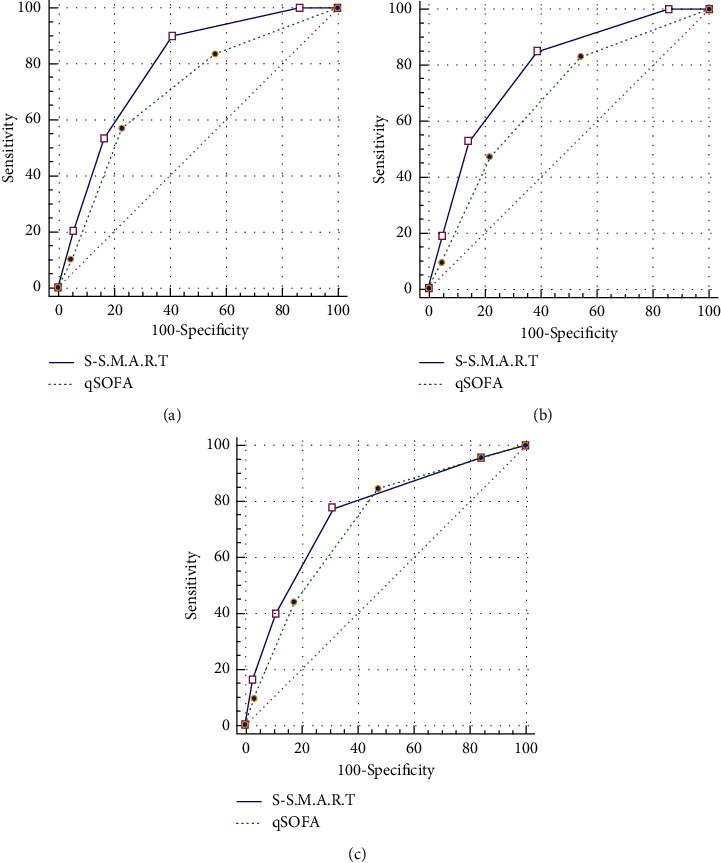
Comparison of receiver operating characteristics curve for prediction of prognosis in patients with sepsis between S-S.M.A.R.T and qSOFA. The S-S.M.A.R.T demonstrated superior performance compared to qSOFA (AUC of 0.789 vs. 0.699; *p*=0.02 for 7-day mortality (a), AUC of 0.786 vs. 0.681; *p* < 0.001 for 30-day mortality (b), and AUC 0.758 vs 0.717; *p*=0.05 for ICU admission (c)). Abbreviations: S-S.M.A.R.T (sepsis evaluation with shock index, mental status, age, and ROX index on triage), qSOFA (quick sequential organ failure assessment), AUC (area under the curve), and ICU (intensive care unit).

The S-S.M.A.R.T had an area under the ROC curve (AUC) of 0.789 (95% confidence interval (CI) 0.746–0.828) for predicting 7-day mortality in patients with suspected sepsis, whereas the qSOFA score had an AUC of 0.699 (95% CI 0.652–0.744) (*p*=0.02). The AUC value for predicting 30-day mortality was 0.786 (95% CI, 0.742–0.825) for the S-S.M.A.R.T and 0.681 (95% CI, 0.633–0.726) for qSOFA (*p* < 0.001). For ICU admission, the AUC for the S-S.M.A.R.T and qSOFA were 0.758 (95% CI, 0.713–0.800) and 0.717 (95% CI, 0.670–0.761), respectively (*p*=0.05) (Figure 2). The sensitivity, specificity, negative predictive value, and positive predictive value for 7-day and 30-day mortality using the S-S.M.A.R.T and qSOFA are presented in Table 3.

The 7-day mortality rates based on the S-S.M.A.R.T scores were 0.0%, 1.7%, 10.8%, 19.6%, and 23.1% for scores of 0, 1, 2, 3, and 4, respectively (*p* < 0.001). The 7-day mortality rates based on qSOFA scores were 3.0%, 6.1%, 17.3%, and 15% for scores of 0, 1, 2, and 3, respectively (*p* < 0.001). The 30-day mortality rates based on the S-S.M.A.R.T scores were 0.0%, 4.7%, 16.7%, 35.3%, and 38.5. % for scores of 0, 1, 2, 3, and 4, respectively (*p* < 0.001), and those based on the qSOFA score were 5.4%, 14.4%, 24.7%, and 25.0% for scores of 0, 1, 2, and 3, respectively (*p* < 0.001). The ICU admission rates according to the S-S.M.A.R.T score were 10.0%, 12.2%, 43.1%, 52.9%, and 73.1% for scores of 0, 1, 2, 3, and 4, respectively (*p* < 0.001), and the corresponding rates according to qSOFA were 10.7%, 35.6%, 49.4%, and 55% for scores of 0, 1, 2, and 3, respectively (*p* < 0.001).

## 4. Discussion

The S-S.M.A.R.T had better predictive ability than the qSOFA for 7-day mortality, 30-day mortality, and ICU admission in patients with suspected sepsis in the ED. Various studies have been reported on the prediction of prognosis in patients with sepsis using the qSOFA score. One study reported an AUC of 0.75 (95% CI 0.71–0.78) for qSOFA, whereas another study reported an AUC of 0.65 (95% CI 0.52–0.77) for qSOFA [8, 9]. In our study, the AUC for qSOFA showed an intermediate value compared to those in other studies. However, the S-S.M.A.R.T showed better performance in predicting both 7-day and 30-day mortalities in patients with sepsis.

In addition, the sensitivity qSOFA scores ≥2 for predicting mortality were 29% and 31.4% in two different studies [8, 9]. Similarly, in our study, qSOFA scores ≥2 for predicting mortality demonstrated low sensitivity for 7-day mortality (56.7%). The Surviving Sepsis Campaign does not recommend using qSOFA as a single screening tool because of its poor sensitivity [2]. S-S.M.A.R.T scores ≥2 demonstrated a higher sensitivity for predicting 7-day mortality (90%) compared to the qSOFA scores. A high-sensitivity screening tool can aid in the early identification of high-risk patients.

The reasons for the S-S.M.A.R.T being more accurate than qSOFA in predicting the severity of sepsis are described. First, old age is a significant risk factor for sepsis [10]. Several studies have shown an association between advanced age and poor prognosis in patients with sepsis [11–13]. Older adults tend to have a higher prevalence of chronic comorbidities such as diabetes, heart disease, and respiratory problems [11]. Preexisting problems such as renal or lung disease are commonly associated with an increased susceptibility to sepsis, and they further compromise the ability to fight infections. In addition, the immune system undergoes age-related changes that lead to functional impairments in both cell-mediated and humoral immunity as individuals grow older [12, 13]. These changes place older patients at a higher risk of developing sepsis, contributing to poor outcomes. Furthermore, atypical symptoms may also be present in older patients with sepsis [12, 14]. The febrile response is blunted and nonspecific signs, such as general weakness and poor oral intake, are common in older patients. Consequently, the recognition of sepsis and the initiation of appropriate treatment may be delayed in older patients.

Second, we replaced systolic blood pressure with the shock index for screening. Although systolic blood pressure is an important indicator, it may not capture the complete picture of hemodynamic instability in patients. The shock index emphasizes the physiological dynamics rather than static criteria. This could allow for a more comprehensive assessment of circulatory compromise in patients by considering the relationship between systolic blood pressure and heart rate [15]. Several previous studies that have evaluated the reliability of the shock index have shown its superiority over heart rate or systolic blood pressure alone [16, 17]. In particular, a shock index ≥1.0 has been associated with significantly worse outcomes in patients [18]. This suggests that the shock index could be a useful tool for screening to facilitate the early recognition and evaluation of patients with suspected sepsis in the ED.

Third, we substituted the respiratory rate with the ROX index. The ROX index was used to identify critically ill patients at a risk of requiring mechanical ventilation [19]. Recently, several studies have demonstrated a relationship between the ROX index and sepsis prognosis [20, 21]. The ROX index was derived by dividing SpO_2_ by FiO_2_ and further dividing it by the respiratory rate. Consequently, the ROX index reflects both the respiratory rate and oxygenation status in patients. Respiratory distress, which is characterized by hypoxemia and labored breathing, is a common feature of organ dysfunction during sepsis [22, 23]. This suggests that the ROX index may provide a more thorough assessment of respiratory distress severity in sepsis than respiratory rate alone. Low SpO_2_, high FiO_2_, and an elevated respiratory rate contribute to a lower ROX index. Lee et al. showed that an ROX index ≤10 was a prognostic factor for 28-day mortality in sepsis [21].

In our study, the S-S.M.A.R.T demonstrated high sensitivity for predicting the prognosis of patients with suspected sepsis. A recent study on the prediction of sepsis prognosis using machine learning showed promising results [24]. Nevertheless, clinical judgement continues to be an essential component in the management of sepsis. The S-S.M.A.R.T can serve as a useful screening tool to assist physicians in their clinical judgement during the treatment of patients with suspected sepsis. This study was conducted in the ED. Patients with sepsis are usually initially diagnosed in the ED. However, few studies have focused on screening tools for predicting the prognosis of patients with sepsis in the ED. The S-S.M.A.R.T may provide valuable insights to emergency physicians not only for predicting the prognosis of patients with sepsis but also for initiating time-sensitive sepsis care bundles.

This study had some limitations. The cutoff values of the shock index and ROX index used in this study were somewhat arbitrary. There is no universal agreement on the cut-off values of these indices for predicting the prognosis of sepsis. Therefore, we applied a combination of values verified in previous studies [18, 21]. Although the numbers 1.0 and 10 used as cutoff values have the advantage of being easy to recall, they may not be optimal cutoff values. Further studies are required to derive and validate the optimal cutoff values. Second, this study was also limited because it was a single-center study with a small sample size, which may restrict the generalizability of our results to all patients with sepsis. Furthermore, the retrospective design of this study may introduce the possibility of inherent bias in patient enrolment.

This study has confirmed the feasibility of using S-S.M.A.R.T as an effective screening tool for predicting the prognosis of patients suspected with sepsis in the ED. This study lays the foundation for additional studies on the S-S.M.A.R.T. By triaging high-risk patients using the S-S.M.A.R.T during the initial assessment in the ED, physicians can facilitate the application of the sepsis bundle, which would ultimately help reduce the mortality rate in patients with sepsis.

## 5. Conclusion

The S-S.M.A.R.T score was associated with mortality in patients with sepsis. The S-S.M.A.R.T score can be useful for predicting the prognosis of patients with suspected sepsis in the ED. However, further studies are required to validate the reliability of this method.

## Figures and Tables

**Table 1:** S-S.M.A.R.T (*s*epsis evaluation with *s*hock index, *m*ental status, *a*ge, and *ROX* index on *t*riage).

Assessment	S-S.M.A.R.T
Shock index (HR/SBP, ≥1.0)	1
Mental status (GCS ≤14)	1
Age (≥65 years)	1
Rox index (SpO_2_/FiO_2_/RR, ≤10)	1

Abbreviations: HR (heart rate); SBP (systolic blood pressure); GCS (Glasgow coma scale); SpO_2_ (saturation of peripheral oxygen); FiO_2_ (fraction of inspired oxygen); RR (respiratory rate).

**Table 2:** Baseline characteristics of the patient.

	Total number of patients (*n*, %)
Age (yr)	72.2 ± 15.6
Gender (F)	213 (53.1%)
*Infection focus*	
Respiratory	161 (40.1%)
Genitourinary	134 (33.4%)
Gastrointestinal	81 (20.2%)
Soft tissue	8 (2.0%)
Other	17 (4.2%)
Bacteremia	159 (39.7%)
qSOFA	
0	168 (41.9%)
1	132 (32.9%)
2	81 (20.2%)
3	20 (5%)
S-S.M.A.R.T	
0	50 (12.5%)
1	172 (42.9%)
2	102 (25.4%)
3	51 (12.7%)
4	26 (6.5%)
*Overall prognosis*	
Length of hospital stay (days)	16.4 ± 15.8
Mechanical ventilation	46 (11.5%)
7-day mortality	30 (7.5%)
30-day mortality	53 (13.2%)
ICU admission	116 (28.9%)

Abbreviations: S-S.M.A.R.T (sepsis evaluation with shock index, mental status, age, and ROX index on triage); qSOFA (quick sequential organ failure assessment); ICU (intensive care unit).

**Table 3:** Comparison between S-S.M.A.R.T and qSOFA for predicting the prognosis of patients with sepsis in the emergency department.

	AUC (95% CI)	*p* value	ROC cut-off	Sensitivity (%)	Specificity (%)	PPV (%)	NPV (%)
S-S.M.A.R.T							
7-day mortality	0.789 (0.746–0.828)	<0.001	≥2	90	59	15.1	98.6
30-day mortality	0.786 (0.742–0.825)	<0.001	≥2	84.9	61.5	25.1	96.4
ICU admission	0.758 (0.713–0.8)	<0.001	≥2	77.6	68.8	50.3	88.3
qSOFA							
7-day mortality	0.699 (0.652–0.744)	<0.001	≥2	56.7	77.4	16.8	95.7
30-day mortality	0.681 (0.633–0.726)	<0.001	≥1	83	45.7	18.9	94.6
ICU admission	0.717 (0.670–0.761)	<0.001	≥1	84.5	52.6	42.1	89.3

Abbreviations: S-S.M.A.R.T (sepsis evaluation with shock index, mental status, age, and ROX index on triage), qSOFA (quick sequential organ failure assessment), AUC (area under the curve), ROC (receiver operator characteristic), PPV (positive predictive value), and NPV (negative predictive value).

## Data Availability

The data used to support the findings of this study are available from the corresponding author upon request.
